# Alabama Dental Association

**Published:** 1897-07

**Authors:** J. M. Walker


### PROCEEDINGS.

### Alabama Dental Association.

Reported for the DENTAL REGISTER by Mrs. J. M. Walker.

The twenty-eighth annual session of the Alabama Dental Association was held in Birmingham, April 13–16, 1897. The meetings were held in the Birmingham Dental College, where ample facilities were afforded for all the purposes of the meeting.

The meeting was opened with prayer by the Rev. A. B. Curry. An eloquent address of welcome was delivered by Mr. Terry representing the Mayor of the city, which was responded to by Dr. R. R. Freeman, Nashville, Tenn., in behalf of the dental profession and Dr. Chas. A. Merrill on behalf of the dentists of Birmingham.

The President, Dr. R. A. Rush, Selma, then delivered his annual address, which was referred to a committee and discussed at a latter session.

After roll-call, the Constitution, By-Laws and Code of Ethics were read, as is the annual custom in the Alabama Association. This is done that those who propose to join the Association may have some definite knowledge of what they are doing when they "sign the Constitution and By-Laws" as newly elected members of the Association.

At the next session Dr. Geo. S. Vann, of Gadsden, read a paper on the "Predisposing Causes of Decay." The paper was highly commended by Dr. J. T. Crawford, Nashville, Tenn., which, he said, was on the subject which of all others, appeals most strongly to him. He believes that one of the most prominent factors in the decay of children's teeth is the present school system, whereby children of from five to seven and eight or ten years of age are deprived of the natural healthy open air life and exercise, the nervous system being unduly stimulated at an age when all the energies are demanded for upbuilding the physical structure—converting food material into bone, blood, skin and muscle.

DR. R. C. YOUNG spoke of the saliva as an index to systemic conditions causing decay of the teeth, and the necessity of more thorough education in order to be able to recognize and combat these conditions.

The subject "Causes of Decay" was discussed at length by Drs. J. Y. Crawford, Young, T. M. Allen, R. R. Freeman and others.

DR. J. P. CORLEY then read a paper on antral empyemia, describing some interesting cases under his care. This paper was discussed by Drs. Gordon White, Nashville, Tenn., Freeman, Dr. Ball (one of the oldest practitioners in the State), Dr. Young, E. A. Wilson, Crawford, Crossland and others, many interesting cases being described, with modes of treatment, etc.

At the night session Dr. W. B. Fulton, Birmingham, read a paper on "Systemic Treatment by Dentists," which was discussed by Drs. S. W. Foster, W. W. Westmoreland, Columbus, Miss., J. H. Crossland, T. M. Allen and others.

At the morning session Wednesday, the subject of society finances was discussed in connection with the committee's report of the President's Address and the annual dues raised to three dollars.

DR. J. Y. CRAWFORD gave an interesting lecture on the subject of "Scientific Diagnosis, and its Necessity in the Practice of Dental Surgery."

DR. GORDON WHITE, Nashville, exhibited a number of valuable appliances. He commended as invaluable to him, an all-glass syringe, the piston being of glass ground to make an accurate fit that no washers are required, thus rendering it easy to utilize the syringe. The syringe is made by A. Wulfing Luer, No. 6 Rue Antoine Dubois, Paris, France, and can also be obtained from W. C. Georgen, 32 East 23rd Street, New York. He showed also a very homely but useful appliance for dressing spatulas, flat burnishers, etc. This is simply a piece of very thick sole leather in which gashes are cut in which pumice is sprinkled; a pair of very delicate scalers for removing deposits on the sides of the roots between the teeth; a sickle-shaped burnisher handy for use on the palatine side of the tooth, or the lingual side of the lower teeth.

Dr. L. P. Haskell described the operations of Dr. Younger in the treatment of pyorrhea alevolaris, in implantation and in regulating teeth by the use of silk ligatures only. He said it was worth going a long distance merely to see Dr. Younger handle these ligatures. In reply to questions, Dr. Haskell stated that Dr. Younger's fee for implantation was $100 a tooth, and for the treatment of pyorrhea $25 an hour.

At the afternoon session the method of dealing with retained decidious and non-erupted permanent teeth, was discussed by Drs. Corley, Allgood, Crawford and others. Dr. Crawford explained the deficient eruption of the deciduous cuspids as being often due to lack of space, the second baby molar in its development crowding the first molar forward and pressing the cuspid back. The permanent cuspids are sometimes caught in the same way between the permanent lateral and the first bicuspid.

The subject of cataphoresis for obtunding sensitive dentine, removal of living pulps, and tooth bleaching, was discussed at some length.

In the discussion of root canal fillings, Dr. R. C. Young spoke very highly of salol with gutta-percha points. Salol has valuable antiseptic qualities, and makes a very perfect root canal filling, as tested by fillings in roots embedded in plaster.

Dr. R. R. Freeman fills the canals with bees-wax, melted in with the Evan's Root Dryer. In case of abcess after root canal filling he drills into the apex from the outside and establishes drainage without any further trouble.

Dr. Crawford spoke of the importance of an accurate knowledge of tooth-anatomy; the number of canals and their location, the difference in the upper and lower posterior teeth, etc. He said that it made but little difference with what the canal was filled provided that it was made aseptic and the apex perfectly sealed. His own method is to carry down to the apex a little of the powder of one of the cements mixed with beechwood creosote. A piece of sterilized orange wood, wrapped with a ribbon of Abbey's gold foil is then driven into the canal which it will fill perfectly. He always requires his patients to come back to him if there is any subsequent trouble, and as they do not come back he concludes that his work is successful. Of all the operations in minor surgery demanding local anesthesia, the removal of a live pulp most demands it. Having decided through diagnosis by exclusion that it is clearly a case of pulpitis and that the pulp must be removed, put on the rubber dam, dry the cavity, wipe it out with bi-chloride of mercury 1–3000, dry again and repeat several times. When satisfied that it is sterilized peel away the dentine carefully until you get a tiny exposure, then take a little muriate of cocaine, 10 percent solution on a tiny wad of cotton and lay it over the point of exposure. With your fingers form of wax a lid for the cavity of the tooth, fit it over and convert the tooth into a force pump, forcing in the cocaine. Then trim away more dentine, enlarge the exposure, and work your pump again, forcing in more cocaine, you will find that you can remove the pulp painlessly and pull it clear out of the lingual canal, which will offer no resistance. Now before going any further with the sterilized orange wood peg, cork up the canal to prevent any debris entering. Now work in more cocaine, and with a broach remove the pulp from the next canal and fill that up, and so on. That tooth is worth all the pains you can take with it and you want to keep the canals in an aseptic condition, preventing the entrance of any debris from the dentine you will remove in preparing the cavity for filling. That pumping process will enable you to anesthize the pulp so that you can remove it without pain to the patient, and you will not want any more arsenic. In case of hemorrhage the application of the bi-chloride of mercury solution will arrest it at once.

If after your careful examination you believe that the pulp is not so diseased as to require removal, that the prognosis is favorable for conservative treatment, even though the tooth is aching. How will you decide the question? Let me tell you, and this is one of the best thoughts of my life! Put in the rubber dam, dry the root and remove the decay; note carefully the entire field of dentine exposed by decay and your operative proceedure; if the dentine is hyper-sensitive or normally sensitive throughout the entire field, you may hope to conserve the vitality of the pulp unless something is wrong in your operative proceedure. But if in the corner nearest the point of exposure the dentine shows opacity and is not sensitive on that side, while it is sensitive in other portions, you may know that that side has been profoundly impressed by the onslaught of inflammation; that that portion of the pulp is not performing its function. Get blood at the point of exposure and as the inflammation is reduced, you will note the dentine becoming more and more sensitive. This is a point of the greatest importance in deciding whether or not to try and save the pulp alive. The contents of the tubules may be only paralyzed and temporarily inactive, but if you fail to get a ready response as above, it is more prudent to wait, but if there is no degenerated portion of the pulp you may hope that nature will resume her functions; that you may get mastery of the disease—convalescence--recovery, and the vitality of the tooth be preserved as though the pulp had never been exposed.

The best application for pulpites is vigorous refrigeration--turn on a stream of cold water until there is immunity from pain.

Dr. R. C. Young: Blind abcess was formerly about the worst thing we had to contend with, but it has lost its terrors. Spray with chloride of ethyl, and go right in, even if there is great inflammation the pain will soon be dispelled, and circulation re-established. Go right in and establish drainage.

At the night session Dr. D. J. McMillin, Kansas City, Mo., gave an illustrated lecture on fractures of the Maxilla, using Angles' appliances as shown by charts and plaster casts with the appliances in position. In the discussion which followed, Dr. Crawford said he had devised a method which he believed would simplify the treatment of fractures. This was the use of staples of platinized iridium, with the ends barbed. He believed they would hold the ends of the fractured bone firmly in position. The only question in his mind was whether the metal might not prove irritating to the tissues. The surgeon in charge of the hospital has agreed to try it in a fracture of one of the long bones. His idea is to split the periosteum and imbed the staple in the bone and leave it in permanently.

DR. J. H. MCCARTY, M. D., read a paper entitled, "Anesthetics, their Use, Danger and Manner of Causing Death." The paper was withheld from publication by the author.

DR. L. P. HASKELL, Chicago, gave a lecture descriptive of his clinic on continuous gum, which was very highly appreciated and constituted the principle feature of the clinics of the meeting.

DR. J. C. WILKERSON showed numerous models of his method of spreading the lower arch by the use of a watch spring.

DR. D. J. MCMILLEN gave a lecture illustrated by charts on the use of non-cohesive gold, which was discussed at some ength.

DR. J. H. CROSSLAND read an essay entitled, "A Dentist's Revery."

DR. W. E. WALKER, Pass Christian, Miss., contributed a brief paper describing the new methods in plate work of Dr. T. C. West of Natchez, as given by him in clinic at the Mississippi meeting. This completed the work of the meeting. The election of officers resulted as follows: Dr. J. H. Crossland, Montgomery, President; Dr. Geo. S. Vann, Gadsden, First Vice-President; Dr. Naftel, of Naftel, Second Vice-President; Dr. Wm. J. Reynolds, Selma, Secretary; Dr. E. M. Rouman, Selma, Treasurer.

Montgomery was selected as the next place of meeting, the time being the second week in April, 1898.

---

FISH bones can be removed from the throat by giving from four to six ounces of milk, and forty minutes later an emetic dose of sulphate of zinc. The coagulated milk carries the bone out with it.—*General Practitioner*.

